# Development of Visceral and Subcutaneous-Abdominal Adipose Tissue in Breastfed Infants during First Year of Lactation

**DOI:** 10.3390/nu13093294

**Published:** 2021-09-21

**Authors:** Zoya Gridneva, Alethea Rea, Ching Tat Lai, Wan Jun Tie, Sambavi Kugananthan, Kevin Murray, Peter E. Hartmann, Donna T. Geddes

**Affiliations:** 1 School of Molecular Sciences, The University of Western Australia, Crawley, WA 6009, Australia; ching-tat.lai@uwa.edu.au (C.T.L.); ash.tie@uwa.edu.au (W.J.T.); samba_k94@hotmail.com (S.K.); peter.hartmann@uwa.edu.au (P.E.H.); donna.geddes@uwa.edu.au (D.T.G.); 2 Mathematics and Statistics, Murdoch University, Murdoch, WA 6150, Australia; alethea.rea@murdoch.edu.au; 3 School of Population and Global Health, The University of Western Australia, Crawley, WA 6009, Australia; kevin.murray@uwa.edu.au

**Keywords:** visceral fat, subcutaneous-abdominal fat, abdominal adiposity, human milk, lactation, infants, body composition, intake, macronutrients, obesity

## Abstract

This study aimed to investigate relationships between infant abdominal visceral and subcutaneous adiposity and human milk (HM) components and maternal body composition (BC) during first year of lactation. Subcutaneous-abdominal depth (SAD), subcutaneous-abdominal fat area (SFA), visceral depth (VD) and preperitoneal fat area of 20 breastfed infants were assessed at 2, 5, 9 and 12 months using ultrasound. Maternal BC was determined with bioimpedance spectroscopy. HM macronutrients and bioactive components concentrations and infant 24-h milk intake were measured and calculated daily intakes (CDI) determined. Maternal adiposity associated with infant SFA (negatively at 2, 5, 12, positively at 9 months, all overall *p* < 0.05). 24-h milk intake positively associated with infant SAD (*p* = 0.007) and VD (*p* = 0.013). CDI of total protein (*p* = 0.013), total carbohydrates (*p* = 0.004) and lactose (*p* = 0.013) positively associated with SFA. Lactoferrin concentration associated with infant VD (negatively at 2, 12, positively at 5, 9 months, overall *p* = 0.003). CDI of HM components and maternal adiposity have differential effects on development of infant visceral and subcutaneous abdominal adiposity. Maintaining healthy maternal BC and continuing breastfeeding to 12 months and beyond may facilitate favourable BC development reducing risk of obesity.

## 1. Introduction

Abdominal obesity is one of the facets of metabolic syndrome and is critically important in clinical diagnosis [1]. Fat distribution in this area plays an important role in outcomes such as obesity-related morbidity and cardio metabolic risk with visceral adipose tissue being the most metabolically and pro-inflammatory active fat depot [2], whilst subcutaneous adipose tissue, specifically superficial subcutaneous adipose tissue, associating with protection [3]. During childhood, accelerated gain in fat mass (FM) [4] and particularly abdominal visceral fat (VF) [5,6] are considered to be a risk factor for metabolic diseases later in life as VF in early life tracks into childhood and beyond [7,8,9]. Furthermore, early sources of nutrition such as breastfeeding are implicated in development of infant body composition (BC) [10] potentially contributing to reported associations of breastfeeding and human milk (HM) with reduced risk of obesity and lower incidence of late metabolic diseases, such as type 2 diabetes [11], although mechanisms of this protection are poorly understood.

Ultrasound has been recently validated to measure infant abdominal visceral and subcutaneous adipose tissue, providing a non-invasive technique for determination of the development of these fat depots during infancy [12]. One study of breastfed and formula-fed infants during the first 6 months of life showed no significant difference in abdominal fat distribution (subcutaneous and preperitoneal fat thickness) between the groups [13]. Contrarily, another study reported that visceral depths were lower at 3 and 12 months of age in infants that were exclusively breastfed at 3 months, suggesting visceral and subcutaneous-abdominal adiposities may be differentially regulated during infancy [12]. Furthermore, the duration of exclusive breastfeeding was found to relate positively to percentage fat mass (%FM) and subcutaneous, but not VF [14], with maternal factors having no effect on development of these fat depots; however, infants were followed up to 6 months only, with 38% exclusively breastfed at 3 months of age (19% at 6 months). The only study that investigated effect of HM macronutrient composition on infant abdominal adiposity showed positive relationships between HM fat concentration and infant abdominal subcutaneous fat thickness, but negative between protein concentration and VF thickness [15].

It is still not understood how breastfeeding and particularly the doses (daily intakes) of HM components influence the acquisition and distribution of abdominal subcutaneous and VF in infants that are breastfed on demand beyond the exclusive breastfeeding period. Establishing the modifiable determinants that influence adiposity and FM development in infancy can inform the support of optimal infant development.

The main aim of this pilot longitudinal study was to evaluate relationships of HM macronutrients (casein, total and whey protein, total carbohydrates and lactose), appetite hormones (adiponectin and leptin) and immune factors (lactoferrin, lysozyme, secretory IGA (sIgA) and HM oligosaccharides (HMO; calculated)) that are present in HM in substantial quantities, with development of abdominal subcutaneous and VF depots in healthy term breastfed infants during the first 12 months of lactation. We also investigated the relationships of these fat depots with maternal BC and with infant 24-h milk intake and breastfeeding frequency.

## 2. Materials and Methods

### 2.1. Subjects and Design

**Figure A1:**
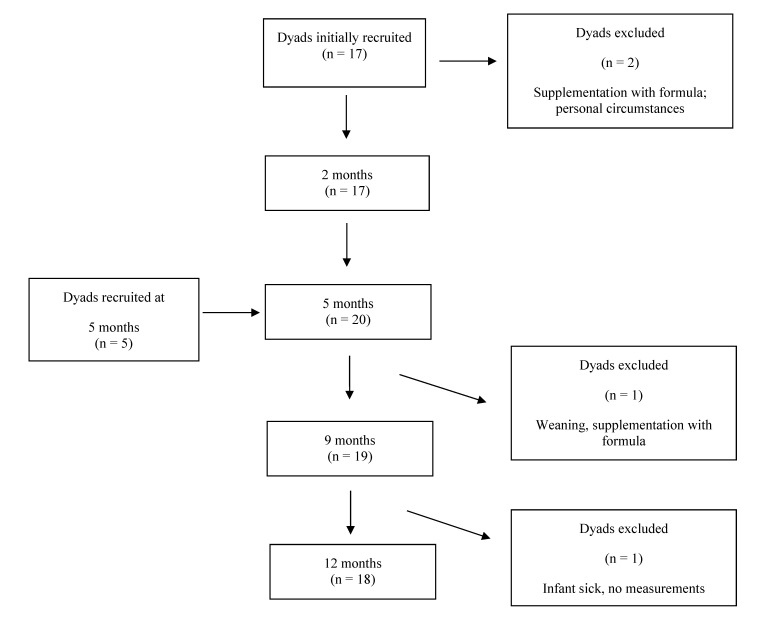
Flowchart of study participants.

This longitudinal observational study included 20 healthy term infants and their mothers who exclusively breastfed to 5 months and continued breastfeeding on demand until 12 months. Mothers were recruited from the community and visited our research laboratory at King Edward Memorial Hospital for Women (Subiaco, Perth, WA, Australia) between March 2013 and September 2015 for up to four measurement sessions (2, 5, 9 and 12 months postpartum) (Figure A1). Eligibility criteria: healthy singletons, gestational age ≥37 weeks, exclusively breastfed to 5 months, breastfed at 9 and 12 months. Exclusion criteria: infant health issues potentially influencing growth and development, formula supplementation at any time, maternal low milk supply and smoking. Women provided written informed consent for participation in the study that was approved by The University of Western Australia Human Research Ethics Committee (RA/4/1/4253, RA/4/1/2639) and registered with Australian New Zealand Clinical Trials Registry (ACTRN12616000368437).

Milk samples were collected and infant abdominal adiposity together with maternal anthropometrics and BC were determined when the infants were 2, 5, 9 and 12-month-old. Dyads were healthy at the time of the visit (no infectious illness, such as cold or flu, no indication of mastitis in mothers at the time of study visit). 24-h milk intake and breastfeeding frequency (meals/24-h) were determined by mothers at home using infant test-weighing before and after breastfeeding between 2 and 5 months, when lactation is stable, and again within 2 weeks of 9 and 12 months [16,17]. Mothers also self-reported breastfeeding frequency at the study visits as the current typical time (hours) between the meals.

### 2.2. Measurements of Infant Abdominal Adiposity

To assess infant inter-abdominal and subcutaneous-abdominal adiposity, single ultrasound measurements were taken with minimal compression with the Aplio XG (Toshiba, Tokyo, Japan) machine and a high-resolution 14–8 MHz transducer (PLT-1204BX) and sterile water-based Aquasonic 100 US transmission gel (Parker Laboratories Inc., Fairfield, NJ, USA). Infants were in supine position for the measurements.

The thicknesses of the preperitoneal and subcutaneous fat (visceral depth (VD) and subcutaneous-abdominal depth (SAD)) were measured in the upper abdomen of infants using a previously validated protocol [12]. For both depths the transducer was positioned where the mid sagittal line intercepts the waist circumference measurement plane, measurements were taken during expiration. VD was measured in the longitudinal plane (probe depth 9 cm) and was determined as the distance between the corpus of the lumbar vertebra and the peritoneal boundary. SAD was measured at the same location, but in the transverse plane (probe depth 4 cm), and determined as the distance between the linea alba and the cutaneous boundary. Depths were measured directly from the images on the screen using electronic calipers. Ratios of VD to SAD (VD/SAD) were calculated.

The areas of the subcutaneous and preperitoneal fat (subcutaneous-abdominal fat area (SFA) and preperitoneal fat area (PFA)) were measured using a standardized protocol for infants [18]. This method has been validated against computed tomography and is a valid method for epidemiological studies [19]. It accounts for age specific differences in infant anatomy, not only measuring the thickness of the fat layers but introducing areas of specific fat depots, ratios of which are highly correlated with metabolic profile in adults [20]. A linear transducer was positioned perpendicular to the skin surface on the median upper abdomen, scans were conducted longitudinally from the xiphoid process to the navel along the linea alba. Distances from linea alba and (a) to the peritoneum on top of the liver, and (b) to the inner surface of subcutaneous tissue were measured directly from the images on the screen using electronic calipers to calculate the SFA and PFA of 2 cm length along the midline, starting from the reference point in direction of navel. Ratios of PFA to SFA (PFA/SFA) were calculated. All of the measurements were performed by one experienced sonographer with previously reported high intra-rater reliability [21].

### 2.3. Measurements of Maternal Body Composition

Maternal BC (fat-free mass (FFM), FM, %FM) was measured with bioelectrical impedance spectroscopy with the Impedimed SFB7 bioelectrical impedance analyzer (ImpediMed, Brisbane, QLD, Australia) according to the manufacturer’s instructions as described previously [22]. The within participant coefficient of variation (CV) for maternal %FM of 0.21% has been reported previously [23]. We also calculated the indices of mothers height-normalized BC: FFM index (FFMI) as FFM/length^2^; FM index (FMI) as FM/length^2^, expressed as kg/m^2^ [24].

### 2.4. Measurements of Human Milk Components

The fully described methods along with measured concentrations, CDI and assays’ detection limits and inter-assay coefficients of variation have been published previously [23,25,26,27,28,29]. In total, we have assessed eleven HM components.

Briefly, pre- and post-feed samples were pooled and defatted for measuring HM components [30] when required by the method with the exception of adiponectin [23] and leptin [25], which were measured in whole milk using ELISA. Casein and whey proteins were separated as described by Kunz and Lonnerdal [31] and Khan et al. [32]. Concentrations of total protein, casein, and whey protein were measured using the Bradford Protein assay [33]. Lactose was measured in pre- and post-feed samples using the enzymatic-spectrophotometric method [33] and averaged for analysis. To determine total carbohydrates (TCH) skim HM was deproteinized with trichloroacetic acid [34] and dehydrated by sulfuric acid [35]. Lysozyme was measured by a modification of Selsted and Martinez method [36,37], and lactoferrin and sIgA were determined by ELISA [37,38]. Standard assays were adapted for and carried out using a JANUS workstation (PerkinElmer, Inc., Waltham, MA, USA) and measured on EnSpire (PerkinElmer, Inc., Waltham, MA, USA). All measurements were conducted in duplicate and averaged for analysis. The total HM oligosaccharide (HMO) concentration was estimated by deducting lactose concentration from TCH concentration.

24-h milk intake values from the 24-h test-weighing [16], and component concentrations measured in HM samples taken at the study sessions were used to determine calculated daily intakes (CDI), considered representative of a typical daily intake for that time point.

### 2.5. Statistical Analyses

The design for the study along with statistical analyses have been previously described in detail [22,26,27,28]. In this longitudinal study, milk samples were collected and infants were measured at 2, 5, 9 and 12 months. An approximate sample size of 14 participants, that would give study power of 0.80 for detection of effect size of 0.15, was calculated in G*Power [39] as for a cross-sectional study, since there is no closed form expression suitable for the calculation of sample sizes for this research design [40], with the consideration that longitudinal study design is more powerful than cross-sectional. Recruitment of participants at the 5-month point was shortly introduced, as at 2 months many mothers would not commit to a study that required to breastfeed to 12 months of age, and the sample size revised upwards (Figure A1). 

Given the longitudinal nature of the data, the relationships were analyzed using linear mixed effects models, and all models had a random effect for each participant to account for the structure in the data. Fitted models included (as explanatory variable and response): (a) infant abdominal fat measures and HM components’ concentrations; (b) abdominal fat measures and CDI of HM components; (c) abdominal fat measures and breastfeeding parameters (24-h milk intake, breastfeeding frequency); and (d) abdominal fat measures and maternal BC parameters. To study the changes over time the models included infant age (as a categorical variable) interacted with the explanatory variable of interest as fixed effects. The *p*-value associated with the interaction was examined, and if it was below 0.05 the results were reported for the full model (fixed effects for infant age, the explanatory variable of interest and the interaction); otherwise results were reported for the model fitted without interaction (fixed effects for infant age and the explanatory variable of interest only).

The analyses for systematic differences between measured variables at different time postpartum used linear mixed model with age as a fixed factor and participant as a random factor. Differences between time points were analyzed using general linear hypothesis tests (Tukey’s all pair comparisons).

Missing data was dealt with using available case analysis. A false discovery rate (FDR) adjustment was applied to the subgroupings of results to the interaction *p* value if it was less than 0.05 or to the main effect *p* value [41]. The largest examined *p* value that is significant and all *p* values before that were considered as significant. The adjusted significance levels are reported in the Tables and set at the 5% level otherwise. Descriptive statistics are reported as mean ± standard deviation (SD) and range; modelling results as parameters estimates ± standard error (SE). Statistical analysis and graphics were performed in R 3.1.5.

## 3. Results

### 3.1. Subjects

Participants demographics, maternal and infant BC, anthropometrics and breastfeeding parameters (24-h milk intake, 24-h breastfeeding frequency, self-reported breastfeeding frequency) as well as attrition and missing data have been published recently [22,26,27,28,29]. Demographic, anthropometric, and breastfeeding parameters measured at the study sessions and the sample sizes at each time point are presented in Table 1. The missing data also included: (a) from the 80 expected, due to the missed visits and infant compliance, measurements of SAD (*n* = 12), VD (*n* = 15), PFA and SFA (*n* = 19); (b) from the 60 expected, CDI of casein, whey and total protein, lactose, adiponectin, leptin, lactoferrin and sIgA (*n* = 27); CDI of total carbohydrates and HMO (*n* = 28); and lysozyme CDI (*n* = 30). The missing data were spread across the time points (Table 1, Figure A1).

### 3.2. Longitudinal Changes in Infant Abdominal Adiposity

Longitudinal changes in maternal and infant BC, breastfeeding parameters and HM components concentrations and CDI have been reported previously [22,26,27,28,29]. Infant abdominal adiposity measurements over the first year of lactation are detailed in Table 1. No significant changes between the time points were seen for VD, PFA, SFA and PFA/SFA ratio. Infant SAD was significantly lower at 12 months postpartum compared with 5 months (−1.65 ± 0.52 mm, *p* = 0.007), whilst VD/SAD ratio was significantly higher at 12 months compared with 5 months (8.66 ± 2.58, *p* = 0.004) and lower at 5 months compared with 2 months (−7.46 ± 2.66, *p* = 0.025).

### 3.3. Human Milk Components and Infant Abdominal Adiposity

**Figure 1:**
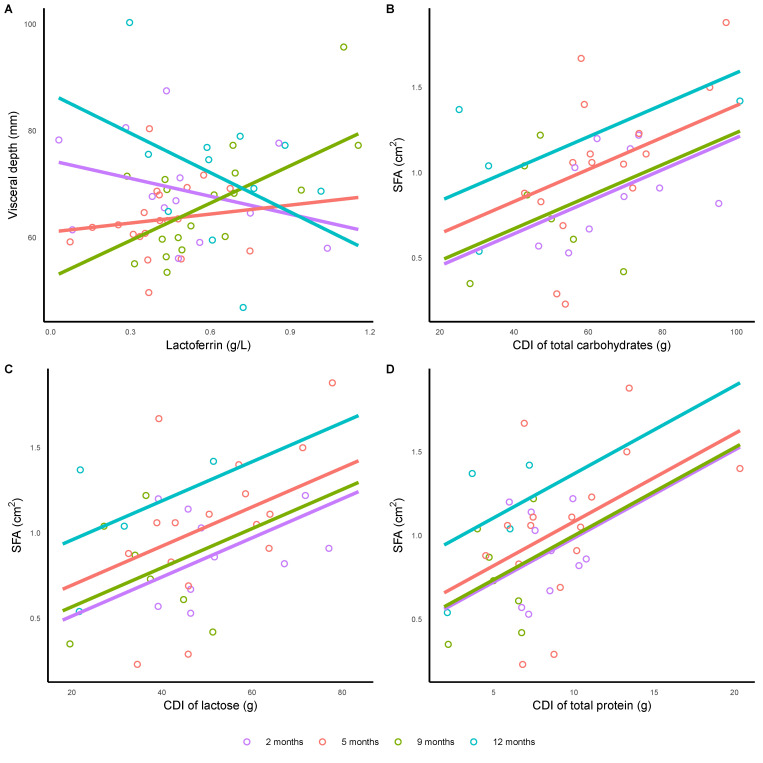
Significant associations between (**A**) infant visceral depth and lactoferrin concentration (*p* = 0.003); and infant subcutaneous fat area (SFA) and calculated daily intakes (CDI) of (**B**) total carbohydrates (*p* = 0.004), (**C**) lactose (*p* = 0.013) and (**D**) total protein (*p* = 0.013). Lines represent linear regression and grouped by the month of lactation.

Higher lactoferrin concentrations were differentially associated with infant VD, lower VD at 2 and 12 months and higher at 5 and 9 months (overall *p* = 0.003), with no other associations for HM components concentrations after the FDR adjustment (Table 2, Figure 1). 

CDI of total protein (*p* = 0.013), total carbohydrates (*p* = 0.004) and lactose (*p* = 0.013) were positively associated with infant SFA (Table 2, Figure 1).

### 3.4. Breastfeeding Parameters and Infant Abdominal Adiposity

**Figure 2:**
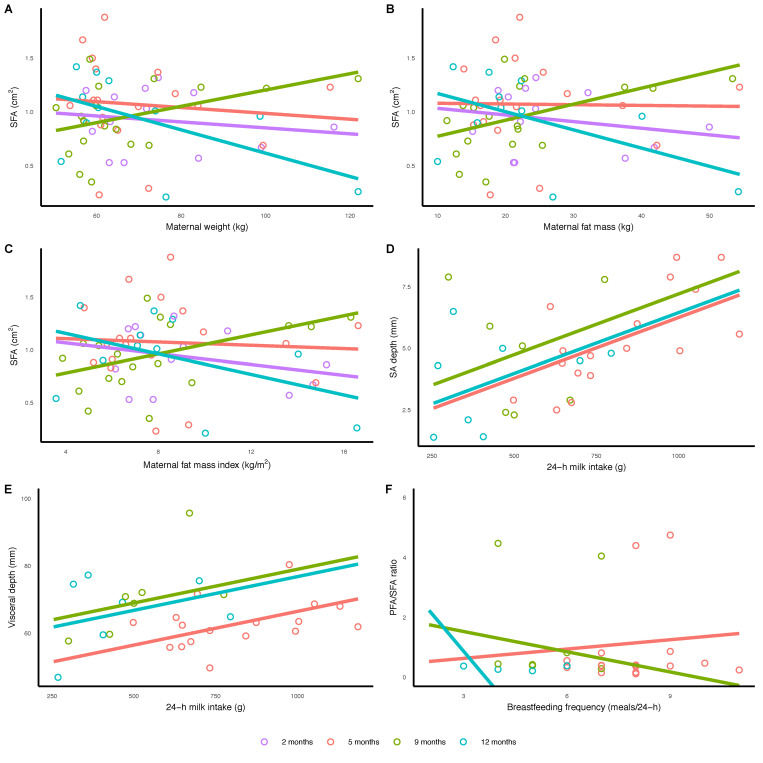
Significant associations between infant subcutaneous fat area (SFA) and (**A**) maternal weight (*p* = 0.020), (**B**) maternal fat mass (*p* = 0.010) and (**C**) fat mass index (*p* = 0.023); 24h milk intake and (**D**) subcutaneous-abdominal (*p* = 0.007) and (**E**) visceral (*p* = 0.013) depths; and (**F**) breastfeeding frequency and peritoneal fat area to subcutaneous fat area ratio (PFA/SFA, *p* = 0.002). Lines represent linear regression and grouped by the month of lactation.

Infant 24-h milk intake was positively associated with infant SAD (*p* = 0.007) and VD (*p* = 0.013). The higher 24-h breastfeeding frequency was associated with infant PFA/SFA ratio; a small increase at 5 months, a small decrease at 9 months and a larger decrease at 12 months (*p* = 0.002). No other associations for breastfeeding parameters were seen after the FDR adjustment (Table 3, Figure 2).

### 3.5. Maternal Body Composition and Infant Abdominal Adiposity

Maternal adiposity (weight, FM, FMI) was negatively associated with infant SFA at 2, 5, 12 months; at 9 months the association was positive (*p* < 0.05), with no other associations for maternal BC after the FDR adjustment (Table 3 and Figure 2).

## 4. Discussion

**Figure 3:**
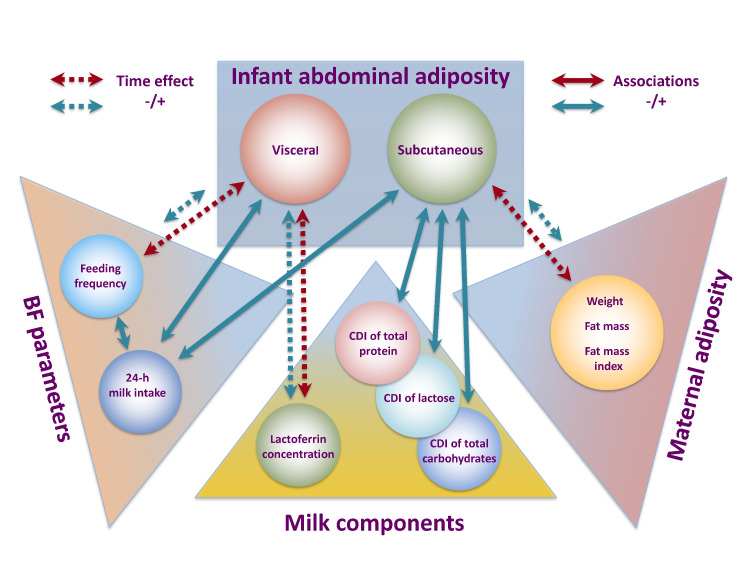
Suggested pathways of lactocrine programming of the infant abdominal adiposity during first 12 months postpartum. Associations between tested parameters and infant abdominal adiposity are indicated by the arrows (green—positive associations; red—negative associations); dotted arrows indicate time-dependent associations. BF—breastfeeding; CDI—calculated daily intake.

This pilot longitudinal study points to the possible complex mechanisms by which HM and breastfeeding may provide some degree of protection from obesity later in life. For the first time we focused on the effect of doses (daily intakes) of an array of HM components on infant abdominal adiposity throughout the first year of breastfeeding on demand. We confirm that in breastfed infant abdominal adipose tissue depots, subcutaneous and visceral, are differentially regulated during infancy. With no longitudinal changes in VF during first 12 months and HM components’ CDI associating predominantly with subcutaneous-abdominal fat depots, these results further inform that breastfeeding and HM do not largely influence VF over the first year of infant nutrition and indeed may be protective against obesity. First and foremost, we link HM composition and components’ intake as well as maternal adiposity and breastfeeding parameters to the development of infant abdominal adiposity (Figure 3).

Reportedly, breastfed infants have higher fat accretion compared with formula-fed [10,42] and non-exclusively breastfed infants [43], speculating that substantial fat accrual during this period of active growth may be protective against obesity later in life. As specific location of fat accretion may determine higher metabolic risk [44], it is imperative to investigate if higher fat accretion in the abdominal area in breastfed infants means more metabolic risk, and if development of these fat depots is independently regulated in this population and affected by nutrition. Few previous studies have investigated the link between infant abdominal adiposity and breastfeeding. One study determined lower VD in 3 and 12-month-old infants that were exclusively breastfed at 3 months [12], although VD measurements in that cohort (2.3–2.8 cm) were unexplainably smaller than ours (6.3–7.1 cm, which relate more to abdominal cavity thickness and are similar to the infant VF thickness measured as distance from the inner face of rectus abdominis muscle to the anterior wall of the aorta (5.0–5.2 cm) [5]). Another study reported duration of exclusive breastfeeding was not associated with VF but was positively related to subcutaneous-abdominal fat at 3 and 6 months and %FM at 6 months [14]. The third study showed no difference in both subcutaneous-abdominal and preperitoneal fat thicknesses between breastfed and formula-fed infants [13]. The most recent study investigated the effect of HM macronutrient composition reporting positive relationships between HM fat concentration (measured in post-feed sample which is the most varied between women and feeds) and infant subcutaneous-abdominal fat thickness, and negative between protein concentration and VF thickness [15]. These results suggest independent regulation of the visceral and subcutaneous-abdominal fat depots in this population.

Although important factors, the presence and duration of any breastfeeding and exclusive breastfeeding are categorical variables usually self-reported by parents. We have measured the actual milk intakes and daily intakes of HM components that infants received with milk to assess the dose effect of these components on development of infant abdominal adiposity. Whilst there are no studies of such design, our findings of time-dependent differential effects of daily intakes of HM macronutrients and bioactive proteins together with breastfeeding parameters further explain the effect of breastfeeding on infant adipose tissue depots.

In line with our previous studies, we found very few associations for the concentrations of HM components, with the only lactoferrin concentration association with infant VD (negative at 2 and 12 months and positive at 5 and 9 months) remaining after multiple comparisons adjustment (Table 2). This is worthy of attention as we previously did not find any associations between lactoferrin concentration and infant BC [29]. This finding is further strengthened with the elucidation of weak time-dependent associations of lactoferrin CDI with preperitoneal adiposity (prior to FDR; Table 2). These associations were negative at 2 and 9 months and positive at 5 and 12 months. We have previously shown lactoferrin CDI to be negatively associated with infant FFMI throughout the first year of lactation in the same cohort [29] emphasising the importance of lactoferrin in the development of BC in breastfed infants. Bovine lactoferrin-supplemented formula is linked to somewhat contrasting results at various time points during first year of life, showing either positive associations with infant length (4–6 months) and weight (6 months) [45], and both, negative association with weight growth rate in female infants [46] and no association with infant growth parameters [47] during the first 12 months. These results support our findings indicating that lactoferrin, including bovine lactoferrin, may have time-dependent effect on infant growth and adiposity during infancy as well as dose-dependent effect, as previously reported for bovine lactoferrin in in vitro studies [48], in which lactoferrin, depending on the dose, either increased or reduced proliferation of porcine intestinal epithelial cells.

Similar to lactoferrin, adiponectin, lysozyme and sIgA concentrations and CDI of adiponectin and sIgA showed similar time-dependent and predominately negative relationships with abdominal adiposity (prior to FDR, Table 2). Recently we reported weak time-dependent associations between infant whole body adiposity and lysozyme concentration [29] and positive relationships with both, lysozyme CDI [29] and adiponectin CDI [26]. Our findings suggest that these bioactive molecules may have multiple timely functions as well as dose-dependent effects on regulation of development of abdominal fat depots and thus, potentially influence risk of obesity later in life. Further larger studies will elucidate the effect of these bioactive HM components on the development and regulation of infant abdominal adiposity. 

Unlike the bioactive proteins, CDI of the major HM macronutrients were positively associated with infant subcutaneous-abdominal adiposity at all-time points (Table 2), with casein also displaying a weak positive association with both subcutaneous-abdominal adiposity parameters. Previously we have not observed strong associations between HM total protein and infant whole body adiposity, yet reported a positive association with HM casein [27]. Furthermore, higher CDI of total carbohydrates and lactose were positively associated with multiple whole body adiposity parameters in the same cohort [28]. These results are supportive of each other as different BC assessment techniques were used (bioimpedance spectroscopy and ultrasound skinfolds) as well as different anatomical skinfold sites. Our findings indicate that within the normal developmental context of breastfeeding HM major macronutrients promote the development of subcutaneous-abdominal rather than visceral adiposity. This is supported by an earlier study that reported higher %FM in exclusively breastfed infants and higher %FM, subcutaneous but not VF, with longer duration of breastfeeding [14], proposing an advantageous adipose phenotype of breastfed infants, associated with a reduced risk of NCD [44].

The associations of macronutrients CDI with subcutaneous-abdominal adiposity parallel the associations of infant breastfeeding parameters and abdominal adiposity. Previously, we have reported that 24-h milk intake was positively associated with infant FM [22]. In the current study, 24-h milk intake also showed positive associations with both, subcutaneous-abdominal adiposity and VD (Table 3). Conversely, breastfeeding frequency, despite being positively related to 24-h milk intake [22], was mainly negatively associated with visceral adiposity (preperitoneal fat area measurements), supporting the necessity of breastfeeding on demand.

Maternal adiposity has been proposed to impact infant adiposity via several mechanisms during both pregnancy [49] and lactation [50]. Our study showed that higher maternal weight and adiposity are associated negatively with infant subcutaneous adiposity (Table 3), suggesting that higher maternal adiposity may limit the protective effect of breastfeeding against obesity later in life. One recent study reported no associations of maternal adiposity with 3-6-month-old infants’ abdominal adiposity, both subcutaneous and visceral, but only proxy adiposity measurements, maternal pre-pregnancy BMI and weight gain, were used in analysis [14]. Whilst participants in our study displayed similar variations in BMI to the previous study, we followed the dyads for a longer period and measured actual maternal BC. 

This pilot study focused on dyads that were breastfeeding on demand for 12 months, thus is more reflective of normal lactation and development of infant abdominal adiposity. The strong points of this study are the longitudinal measurements of dyads and sampling of HM, accounting for the dose (intake) of HM components, measuring maternal BC and the wide variation in maternal adiposity. The limitations are the modest sample size associated with the multiple measurement time points and the small number of 24-h milk intake measurements at 9 and 12 months postpartum. The preperitoneal fat measurements are the proxy estimations of VF and may be prone to higher measurement error, leading to underestimated associations. A substantial accrual of preperitoneal fat occurs after 2 years of age [18], so measurements and associations of preperitoneal fat in our study may not necessarily be predictive of abdominal adiposity later in life. Our sample is relatively homogenous (predominantly Caucasian, term, healthy breastfed singletons from urban mothers of higher social-economic status), thus our results may not be transferable to populations that are more diverse.

## 5. Conclusions

The findings from this pilot study highlight the complex mechanisms by which breastfeeding and HM may provide some degree of protection from obesity and reduce risk factors for NCD later in life. The time-dependent differential associations of daily intakes of HM macronutrients and bioactive proteins, as well as breastfeeding parameters and maternal adiposity, with the development of infant abdominal adiposity during the first year of life indicate a potential to improve infant outcomes via interventions, such as extending the duration of breastfeeding to the first year and presumably beyond, and improving maternal BC pre-conception and during pregnancy and lactation.

## Figures and Tables

**Table 1:** Participant anthropometric, abdominal adiposity and breastfeeding characteristics.

Characteristics	2 Months	5 Months	9 Months	12 Months
	Mean ± SD (Min–Max)	Mean ± SD (Min–Max)	Mean ± SD (Min–Max)	Mean ± SD (Min–Max)
Mothers	(*n* = 14)	(*n* = 20)	(*n* = 18)	(*n* = 18)
Weight (kg)	78.8 ± 19.3 ^a^	70.1 ± 17.8	63.0 ± 10.0	64.2 ± 17.3
	(57.5–116.2)	(53.7–115.3)	(50.4–121.9)	(51.4–121.9)
BMI (kg/m^2^)	27.2 ± 5.5	24.8 ± 5.0	22.7 ± 3.9	23.9 ± 5.9
	(20.4–35.5)	(19.0–35.2)	(17.9–37.2)	(18.2–37.2)
Infants	(*n* = 15)	(*n* = 20)	(*n* = 19)	(*n* = 18)
Sex (M/F)	9 M/6 F	10 M/10 F	10 M/9 F	9 M/9 F
Age (months)	2.04 ± 0.14	5.16 ± 0.22	9.22 ± 0.27	12.26 ± 0.28
	(1.87–2.33)	(4.77–5.47)	(8.83–9.77)	(11.63–12.67)
Length (cm)	58.1 ± 1.9	64.8 ± 2.3	71.7 ± 1.9	73.6 ± 3.2
	(54.2–60.0)	(60.5–69.5)	(66.0–74.0)	(69.0–78.5)
Weight (kg)	5.63 ± 0.66	7.431 ± 1.134	8.836 ± 0.975	9.650 ± 0.618
	(4.420–7.400)	(5.808–9.510)	(6.675–10.095)	(7.165–11.085)
BMI (kg/m^2^)	16.6 ± 1.2	17.6 ± 1.9	17.7 ± 1.7	17.8 ± 0.9
	(14.5–18.1)	(14.9–20.4)	(14.2–20.2)	(13.7–19.2)
VD (mm)	68.8 ± 9.7	63.3 ± 6.7	66.9 ± 10.3	71.3 ± 12.3
	(56.1–87.5)	(49.7–80.4)	(53.5–95.7)	(46.9–100.3)
SAD (mm)	4.0 ± 1.8	5.3 ± 1.8 **	4.6 ± 2.1	3.7 ± 1.9 **
	(1.5–7.2)	(2.5–8.7)	(2.1–8.4)	(1.4–8.5)
VD/SAD ratio	22.0 ± 14.2 *	13.0 ± 5.0 *^,^**	18.0 ± 8.8	22.6 ± 11.5 **
	(8.6–58.3)	(7.0–25.9)	(7.1–33.0)	(9.1–42.2)
SFA (cm^2^)	0.9 ± 0.3	1.1 ± 0.4	0.9 ± 0.3	0.9 ± 0.4
	(0.5–1.3)	(0.2–1.9)	(0.4–1.5)	(0.2–1.4)
PFA (cm^2^)	0.3 ± 0.1	0.5 ± 0.3	0.5 ± 0.5	0.4 ± 0.1
	(0.2–0.4)	(0.2–1.3)	(0.3–1.8)	(0.2–0.7)
SFA/PFA ratio	0.3 ± 0.1	0.8 ± 1.3	0.9 ± 1.4	0.6 ± 0.7
	(0.1–0.5)	(0.2–4.8)	(0.2–5.1)	(0.2–2.7)
Age (months)	2.04 ± 0.14	5.16 ± 0.22	9.22 ± 0.27	12.26 ± 0.28
	(1.87–2.33)	(4.77–5.47)	(8.83–9.77)	(11.63–12.67)
Breastfeeding characteristics				
24-h milk intake (g)	n/a ^b^	(*n* = 17)	(*n* = 8)	(*n* = 8)
		818.8 ± 204.9	478.3 ± 154.0	451.1 ± 215.7
		(498–1185)	(300–775)	(255–795)
24-h feeding frequency (24-h MP)	n/a ^b^	(*n* = 17)	(*n* = 8)	(*n* = 9)
		8.1 ± 1.4	5.4 ± 1.3	4.4 ± 2.1
		(6–11)	(4–7)	(2–8)
Feeding frequency (SR) ^c^	(*n* = 11)	(*n* = 19)	(*n* = 17)	(*n* = 13)
	2.3 ± 0.4	2.8 ± 0.8	3.7 ± 1.2	5.4 ± 2.9
	(1.5–3.0)	(1.5–4.0)	(2.0–6.0)	(2.2–12.0)

BMI, body mass index; M/F, male/female; MP, milk production; n/a not applicable; PFA, preperitoneal fat area; SAD, subcutaneous-abdominal depth; SAF, subcutaneous-abdominal fat area; SR, self-reported; VD, visceral depth. ^a^ Data are mean ± SD and ranges. ^b^ 24-h milk intake and breastfeeding frequency (meals/24-h) were measured between 2 and 5 months (presented at 5 months) and within 2 weeks of 9 and 12 months. ^c^ Maternal self-report (SR) of breastfeeding frequency at the time of the visit as a typical time between meals (e.g., each 2 h). * *p* < 0.05; ** *p* < 0.01 indicate significant differences in infant abdominal adiposity measures between the two marked time points.

**Table 2:** Associations between infant abdominal adiposity parameters and concentrations and daily intakes of human milk components.

Predictor	2 Months		5 Months		9 Months		12 Months		*p* Value		
	Intercept (SE)	Slope (SE)	Intercept (SE)	Slope (SE)	Intercept (SE)	Slope (SE)	Intercept (SE)	Slope (SE)	Predictor	Infant Age (Months)	Interaction
Concentrations	(*n* = 14)		(*n* = 20)		(*n* = 17)		(*n* = 13)		(*n* = 20)		
Subcutaneous-abdominal depth (mm), significance at <0.041 ^d^											
Adiponectin, g/L	5.3 (1.14)	−0.102 (0.098) ^a^	3.19 (1.14)	0.221 (0.112)	5.43 (2.26)	−0.106 (0.254)	5.58 (1.33)	−0.17 (0.112)	0.64	0.038	0.041 ^b^
Visceral depth (mm), significance at <0.019											
Lactoferrin, g/L	74.5 (4.93)	−11.2 (8.85)	61.0 (5.55)	5.61 (12.8)	52.4 (5.49)	23.4 (8.44)	86.9 (8.47)	−24.6 (12.7)	0.70	0.084	**0.003**
Lysozyme, g/L	73.3 (3.06)	−32.5 (14.1)	65.9 (2.65)	−32.5 (14.1)	71.2 (2.96)	−32.5 (14.1)	77.6 (4.03)	−32.5 (14.1)	0.019 ^c^	0.007	0.67
Visceral/subcutaneous-abdominal depths ratio, significance at <0.006											
Total protein, g/L	−16.2 (16.8)	3.55 (1.6)	12.7 (6.61)	0.063 (0.554)	5.6 (11.4)	1.17 (1.08)	48.9 (11.5)	−2.58 (1.07)	0.93	0.003	0.006
Preperitoneal fat area (cm^2^), significance at <0.013											
Total protein, g/L	0.539 (0.622)	−0.024 (0.057)	0.317 (0.254)	0.016 (0.021)	1.96 (0.441)	−0.137 (0.042)	0.43 (0.397)	−0.008 (0.034)	0.40	0.059	0.013
sIgA, g/L	0.378 (0.248)	−0.164 (0.426)	0.462 (0.21)	0.088 (0.388)	1.25 (0.22)	−1.17 (0.341)	0.38 (0.34)	−0.062 (0.50)	0.038	0.045	0.049
Calculated daily intakes	(*n* = 12)		(*n* = 17)		(*n* = 8)		(*n* = 8)		(*n* = 17)		
Subcutaneous-abdominal depth (mm), significance at <0.005											
Adiponectin, g	4.59 (1.12)	−0.00005 (0.0001)	2.69 (0.97)	0.0003 (0.0001)	6.3 (2.06)	−0.0003 (0.0004)	4.05 (2.08)	−0.0001 (0.0005)	0.094	0.079	0.027
Casein, g	2.66 (0.845)	1.34 (0.555)	3.43 (0.928)	1.34 (0.555)	3.98 (0.794)	1.34 (0.555)	2.86 (0.744)	1.34 (0.555)	0.021	0.45	0.91
Lactose, g	1.18 (1.38)	0.054 (0.023)	2.46 (1.35)	0.054 (0.023)	2.9 (1.1)	0.054 (0.023)	2.02 (0.976)	0.054 (0.023)	0.021	0.17	0.27
sIgA, g	4.97 (1.33)	−2.82 (3.34)	4.2 (1.04)	2.97 (2.39)	9.09 (1.76)	−15.1 (5.78)	4.36 (2.33)	−2.36 (8.51)	0.70	0.11	0.029
Total carbohydrates, g	0.524 (1.32)	0.047 (0.018)	2.27 (1.24)	0.047 (0.018)	2.59 (1.08)	0.047 (0.018)	1.67 (1.0)	0.047 (0.018)	0.005	0.042	0.16
Subcutaneous-abdominal fat area (cm^2^), significance at <0.027											
Casein, g	0.632 (0.161)	0.235 (0.103)	0.713 (0.175)	0.235 (0.103)	0.608 (0.148)	0.235 (0.103)	0.951 (0.188)	0.235 (0.103)	0.027	0.45	0.79
Lactose, g	0.283 (0.269)	0.012 (0.005)	0.464 (0.253)	0.012 (0.005)	0.338 (0.211)	0.012 (0.005)	0.729 (0.226)	0.012 (0.005)	**0.013**	0.16	0.36
sIgA, g	0.604 (0.186)	0.742 (0.379)	0.757 (0.177)	0.742 (0.379)	0.551 (0.173)	0.742 (0.379)	0.921 (0.206)	0.742 (0.379)	0.042	0.20	0.44
Total carbohydrates, g	0.266 (0.251)	0.009 (0.003)	0.455 (0.233)	0.009 (0.003)	0.296 (0.473)	0.009 (0.003)	0.647 (0.234)	0.009 (0.003)	**0.004**	0.18	0.20
Total protein, g	0.459 (0.203)	0.053 (0.021)	0.557 (0.214)	0.053 (0.021)	0.473 (0.169)	0.053 (0.021)	0.842 (0.198)	0.053 (0.021)	**0.013**	0.31	0.81
Preperitoneal/subcutaneous-abdominal fat areas ratio, significance at <0.028											
Lactoferrin, g	1.44 (0.828)	−2.52 (2.28)	0.057 (0.813)	2.92 (2.38)	2.23 (0.757)	−2.36 (1.88)	−4.91 (2.24)	14.2 (7.28)	0.52	0.001	0.028

sIgA, secretory immunoglobulin A. ^a^ Data are parameter estimate ± SE (standard error of estimate); effects of predictors taken from linear mixed effects models that accounted for month after birth and an interaction between month after birth and predictor with a random effect for participant; if the *p* value for interaction is not <0.05 parameter estimates are taken from a model with no interaction. ^b,c^ Results are presented only for interactions or predictors with raw *p* values < 0.05. ^d^ Significance for the tested combinations after the false discovery rate adjustment (significant *p* values are indicated by the bold text).

**Table 3:** Associations between infant abdominal adiposity and breastfeeding parameters and maternal body composition.

Predictor	2 Months		5 Months		9 Months		12 Months		*p* Value		
	Intercept (SE)	Slope (SE)	Intercept (SE)	Slope (SE)	Intercept (SE)	Slope (SE)	Intercept (SE)	Slope (SE)	Predictor	Infant Age (Months)	Interaction
Breastfeeding parameters			(*n* = 17)		(*n* = 7)		(*n* = 8)		(*n* = 17)		
Subcutaneous-abdominal depth (mm), significance at <0.005 ^d^											
24-h milk intake, g ^e^	n/a ^e^		1.32 (1.56)	0.005 (0.002) ^a^	2.27 (1.18)	0.005 (0.002)	1.51 (1.04)	0.005 (0.002)	**0.007** ^b^	0.57	0.30
Subcutaneous-abdominal fat area (cm^2^), significance at <0.022											
24-h milk intake, g	n/a		0.295 (0.347)	0.001 (0.0004)	0.252 (0.262)	0.001 (0.0004)	0.641 (0.277)	0.001 (0.0004)	0.022	0.24	0.31
Visceral depth (mm), significance at < 0.05											
24-h milk intake, g	n/a		46.50 (7.35)	0.02 (0.009)	59.00 (5.33)	0.02 (0.009)	56.8 (5.12)	0.02 (0.009)	**0.013**	0.007	0.068
Preperitoneal fat area (cm^2^), significance at <0.047											
Breastfeeding Frequency ^e^	n/a		096 (0.491)	−0.046 (0.059)	1.06 (0.346)	−0.046 (0.059)	0.23 (0.31)	−0.046 (0.059)	0.047	<0.001	0.082
Preperitoneal/subcutaneous-abdominal fat areas ratio, significance at <0.05											
Breastfeeding frequency	n/a		0.37 (2.1)	0.097 (0.257)	2.85 (1.54)	−0.305 (0.276)	4.81 (1.72)	−1.34 (0.372)	0.19	<0.001	**0.002** ^c^
Maternal body composition	(*n* = 14)		(*n* = 20)		(*n* = 18)		(*n* = 17)		(*n* = 20)		
Subcutaneous-abdominal fat area (cm^2^), significance at <0.048											
Fat mass, kg	1.10 (0.276)	−0.006 (0.01)	1.09 (2.03)	−0.0007 (0.008)	0.625 (0.189)	0.015 (0.008)	1.34 (0.221)	−0.017 (0.008)	0.91	0.46	**0.010**
Fat mass, %	1.42 (0.644)	−0.014 (0.018)	0.839 (0.443)	0.007 (0.013)	0.087 (0.393)	0.027 (0.012)	1.62 (0.474)	−0.021 (0.015)	0.57	0.49	**0.019**
Weight, kg	1.13 (0.447)	−0.003 (0.006)	1.26 (0.366)	−0.003 (0.005)	0.446 (0.325)	0.008 (0.005)	1.70 (0.369)	−0.011 (0.005)	0.66	0.49	**0.020**
Fat mass index, kg/m^2^	1.17 (0.308)	−0.026 (0.031)	1.14 (0.227)	−0.008 (0.025)	0.599 (0.209)	0.045 (0.024)	1.35 (0.257)	−0.049 (0.028)	0.88	0.48	**0.023**
Fat-free mass, kg	1.06 (0.657)	−0.003 (0.014)	1.76 (0.60)	−0.015 (0.013)	0.464 (0.489)	0.011 (0.010)	2.21 (0.569)	−0.027 (0.012)	0.39	0.54	0.048
Visceral depth (mm), significance at <0.045											
Fat mass, kg	74.40 (7.47)	−0.201 (0.274)	63.10 (5.45)	0.009 (0.214)	69.20 (5.19)	−0.127 (0.208)	58.30 (5.65)	0.512- (0.225)	0.65	0.077	0.045
Weight, kg	76.60 (11.70)	−0.101 (0.157)	62.40 (9.82)	0.014 (0.14)	68.90 (8.84)	−0.038 (0.127)	48.30 (9.31)	0.319 (0.132)	0.48	0.081	0.049
Preperitoneal/subcutaneous-abdominal fat areas ratio, significance at <0.022											
Fat mass, kg	0.757 (0.853)	−0.012 (0.030)	1.34 (0.639)	−0.020 (0.025)	1.57 (0.595)	−0.032 (0.024)	−0.859 (0.685)	0.053 (0.026)	0.82	0.35	0.022
Weight, kg	0.88 (1.39)	−0.006 (0.018)	1.49 (1.15)	−0.009 (0.016)	1.95 (1.02)	−0.016 (0.015)	−1.81 (1.15)	0.031 (0.016)	0.99	0.37	0.042
Fat mass, %	1.42 (1.98)	−0.029 (0.056)	2.23 (1.39)	−0.041 (0.041)	2.85 (1.23)	−0.063 (0.038)	−2.06 (1.46)	0.076 (0.045)	0.54	0.35	0.045
Fat-free mass index, kg/m^2^	−2.25 (1.37)	0.16 (0.081)	−1.72 (1.32)	0.16 (0.081)	−1.72 (1.32)	0.16 (0.081)	−2.20 (1.39)	0.16 (0.081)	0.046	0.29	0.60

^a^ Data are parameter estimate ± SE (standard error of estimate); effects of predictors taken from linear mixed effects models that accounted for month after birth and an interaction between month after birth and predictor with a random effect for participant; if the *p* value for interaction is not <0.05 parameter estimates are taken from a model with no interaction. ^b,c^ Results are presented only for interactions or predictors with raw *p* values < 0.05. ^d^ Significance for the tested combinations after the false discovery rate adjustment (significant *p* values are indicated by the bold text). ^e^ 24-h milk intake and breastfeeding frequency (meals/24-h) were measured between 2 and 5 months (presented at 5 months) and within 2 weeks of 9 and 12 months.

## Data Availability

The data presented in this study are available from the corresponding author upon reasonable request.

## References

[1] Després J.-P., Lemieux I. (2006). Abdominal obesity and metabolic syndrome. Nature.

[2] Hamdy O., Porramatikul S., Al-Ozairi E. (2006). Metabolic obesity: The paradox between visceral and subcutaneous fat. Cur. Diabetes Rev..

[3] Golan R., Shelef I., Rudich A., Gepner Y., Shemesh E., Chassidim Y., Harman-Boehm I., Henkin Y., Schwarzfuchs D., Ben Avraham S. (2012). Abdominal superficial subcutaneous fat: A putative distinct protective fat subdepot in type 2 diabetes. Diabetes Care.

[4] Leunissen R.W., Kerkhof G.F., Stijnen T., Hok-ken-Koelega A. (2009). Timing and tempo of first- year rapid growth in relation to cardiovascular and metabolic risk profile in early adulthood. JAMA.

[5] Ferreira A.P., da Silva Junior J.R., Figueiroa J.N., Alves J.G. (2014). Abdominal subcutaneous and visceral fat thickness in newborns: Correlation with anthropometric and metabolic profile. J. Perinatol..

[6] Goran M.I., Gower B.A. (1999). Relation between visceral fat and disease risk in children and adolescents. Am. J. Clin. Nutr..

[7] Gesta S., Tseng Y.H., Kahn C.R. (2007). Developmental origin of fat: Tracking obesity to its source. Cell.

[8] Gishti O., Gaillard R., Durmus B., Abrahamse M., van der Beek E.M., Hofman A., Franco O.H., de Jonge L.L., Jaddoe V.W. (2015). BMI, total and abdominal fat distribution, and cardiovascular risk factors in school-age children. Pediatr. Res..

[9] Liu K.H., Chan Y.L., Chan W.B., Kong W.L., Kong M.O., Chan J.C. (2003). Sonographic measurement of mesenteric fat thickness is a good correlate with cardiovascular risk factors: Comparison with subcutaneous and preperitoneal fat thickness, magnetic resonance imaging and anthropometric indexes. Int. J. Obes..

[10] Butte N., Wong W., Hopkinson J., Smith E., Ellis K. (2000). Infant feeding mode affects early growth and body composition. Pediatrics.

[11] Woo J.G., Martin J.M. (2015). Does breastfeeding protect against childhood obesity? Moving beyond observational evidence. Curr. Obes. Rep..

[12] De Lucia Rolfe E., Modi N., Uthaya S., Hughes I.A., Dunger D.B., Acerini C., Stolk R.P., Ong K.K. (2013). Ultrasound estimates of visceral and subcutaneous-abdominal adipose tissues in infancy. J. Obes..

[13] Barros V.O., Amorim M.R., Melo A.O., Tavares J.S., Silva A.C., Alves J.G. (2016). Abdominal fat distribution among breastfed and formula-fed infants. Breastfeed. Med..

[14] Breij L.M., Abrahamse-Berkeveld M., Acton D., De Lucia Rolfe E., Ong K.K., Hokken-Koelega A.C.S. (2017). Impact of early infant growth, duration of breastfeeding and maternal factors on total body fat mass and visceral fat at 3 and 6 months of age. Ann. Nutr. Metab..

[15] de Fluiter K., Kerkhof G.F., van Beijsterveldt I., Breij L.M., de Heijning B., Abrahamse-Berkeveld M., Hokken-Koelega A. (2020). Longitudinal human milk macronutrients, body composition and infant appetite during early life. Clin. Nutr..

[16] Arthur P., Hartmann P., Smith M. (1987). Measurement of the milk intake of breast-fed infants. J. Pediatr. Gastroenterol. Nutr..

[17] Kent J.C., Mitoulas L.R., Cregan M.D., Ramsay D.T., Doherty D.A., Hartmann P.E. (2006). Volume and frequency of breastfeedings and fat content of breast milk throughout the day. Pediatrics.

[18] Holzhauer S., Zwijsen M.L., Jaddoe V.W.V., Boehm G., Moll H.A., Mulder P.G., Kleyburg-Linkers V.A., Hofman A., Witteman J.C.M. (2009). Sonographic assessment of abdominal fat distribution in infancy. Eur. J. Epidemiol..

[19] Mook-Kanamori D.O., Holzhauer S., Hollestein L.M., Durmus B., Manniesing R., Koek M., Boehm G., van der Beek E.M., Hofman A., Witteman J.C. (2009). Abdominal fat in children measured by ultrasound and computed tomography. Ultrasound Med. Biol..

[20] Suzuki R., Watanabe S., Hirai Y., Akiyama K., Nishide T., Matsushima Y., Murayama H., Ohshima H., Shinomiya M., Shirai K. (1993). Abdominal wall fat index, estimated by ultrasonography, for assessment of the ratio of visceral fat to subcutaneous fat in the abdomen. Am. J. Med..

[21] McLeod G., Geddes D., Nathan E., Sherriff J., Simmer K., Hartmann P. (2013). Feasibility of using ultrasound to measure preterm body composition and to assess macronutrient influences on tissue accretion rates. Early Hum. Dev..

[22] Gridneva Z., Rea A., Hepworth A.R., Ward L.C., Lai C.T., Hartmann P.E., Geddes D.T. (2018). Relationships between breastfeeding patterns and maternal and infant body composition over the first 12 months of lactation. Nutrients.

[23] Kugananthan S., Gridneva Z., Lai C.T., Hepworth A.R., Mark P.J., Kakulas F., Geddes D.T. (2017). Associations between maternal body composition and appetite hormones and macronutrients in human milk. Nutrients.

[24] Van Itallie T.B., Yang M.U., Heymsfield S.B., Funk R.C., Boileau R.A. (1990). Height-normalized indices of the body’s fat-free mass and fat mass: Potentially useful indicators of nutritional status. Am. J. Clin. Nutr..

[25] Kugananthan S., Lai C.T., Gridneva Z., Mark P.J., Geddes D.T., Kakulas F. (2016). Leptin levels are higher in whole compared to skim human milk, supporting a cellular contribution. Nutrients.

[26] Gridneva Z., Kugananthan S., Rea A., Lai C.T., Ward L.C., Murray K., Hartmann P.E., Geddes D.T. (2018). Human milk adiponectin and leptin and infant body composition over the first 12 months of lactation. Nutrients.

[27] Gridneva Z., Tie W.J., Rea A., Lai C.T., Ward L.C., Murray K., Hartmann P.E., Geddes D.T. (2018). Human milk casein and whey protein and infant body composition over the first 12 months of lactation. Nutrients.

[28] Gridneva Z., Rea A., Tie W.J., Lai C.T., Kugananthan S., Ward L.C., Murray K., Hartmann P.E., Geddes D.T. (2019). Carbohydrates in human milk and body composition of term infants during the first 12 months of lactation. Nutrients.

[29] Gridneva Z., Lai C.T., Rea A., Tie W.J., Ward L.C., Murray K., Hartmann P.E., Geddes D.T. (2020). Human milk immunomodulatory proteins are related to development of infant body composition during the first year of lactation. Pediatr. Res..

[30] Keller R., Neville M. (1986). Determination of total protein in human milk: Comparison of methods. Clin. Chem..

[31] Kunz C., Lonnerdal B. (1989). Human milk proteins: Separation of whey proteins and their analysis by polyacrylamide gel electrophoresis, fast protein liquid chromatography (FPLC) gel filtration, and anion-exchange chromatography. Am. J. Clin. Nutr..

[32] Khan S., Casadio Y., Lai C., Prime D., Hepworth A., Trengove N., Hartmann P. (2012). Investigation of short-term variations in casein and whey proteins in breast milk of term mothers. Hepat. Nutr..

[33] Mitoulas L.R., Kent J.C., Cox D.B., Owens R.A., Sherriff J.L., Hartmann P.E. (2002). Variation in fat, lactose and protein in human milk over 24 h and throughout the first year of lactation. Br J. Nutr..

[34] Euber J., Brunner J. (1979). Determination of lactose in milk products by high-performance liquid chromatography. J. Dairy Sci..

[35] Albalasmeh A., Berhe A., Ghezzehei T. (2013). A new method for rapid determination of carbohydrate and total carbon concentrations using UV spectrophotometry. Carbohydr. Polym..

[36] Selsted M., Martinez R. (1980). A simple and ultrasensitive enzymatic assay for the quantitative determination of lysozyme in the picogram range. Anal. Biochem..

[37] Zhang G., Lai C.T., Hartmann P., Oddy W.H., Kusel M.M.H., Sly P.D., Holt P.G. (2014). Anti-infective proteins in breast milk and asthma-associated phenotypes during early childhood. Pediatr. Allergy Immunol..

[38] Tijssen P., Burdon R.H., van Knippenberg P.H. (1985). Practice and theory of immunoessays. Laboratory Techniques in Biochemistry and Molecular Biology.

[39] Faul F., Erdfelder E., Buchner A., Lang A.-G. (2009). Statistical power analyses using G*Power 3.1: Tests for correlation and regression analyses. Behav. Res. Methods.

[40] Diggle P.J., Heagerty P.J., Liang K.-Y., Zeger S.L. (2002). Analysis of Longitudinal Data.

[41] Curran-Everett D. (2000). Multiple comparisons: Philosophies and illustrations. Am. J. Physiol. Regul. Integr. Comp. Physiol..

[42] Carberry A., Golditz P., Lingwood B. (2010). Body composition from birth to 4.5 months in infants born to non-obese women. Pediatr. Res..

[43] Rodríguez-Cano A.M., Mier-Cabrera J., Allegre-Dávalos A.L., Muñoz-Manrique C., Perichart-Perera O. (2019). Higher fat mass and fat mass accretion during the first six months of life in exclusively breastfed infants. Pediatr. Res..

[44] Booth A., Magnuson A., Foster M. (2014). Detrimental and protective fat: Body fat distribution and its relation to metabolic disease. Horm. Mol. Biol. Clin. Investig..

[45] Hernell O., Lonnerdal B. (2002). Iron status of infants fed low-iron formula: No effect of added bovine lactoferrin or nucleotides. Am. J. Clin. Nutr..

[46] Johnston W.H., Ashley C., Yeiser M., Harris C.L., Stolz S.I., Wampler J.L., Wittke A., Cooper T.R. (2015). Growth and tolerance of formula with lactoferrin in infants through one year of age: Double-blind, randomized, controlled trial. BMC Pediatr..

[47] King J.C., Cummings G.E., Guo N., Triverdi L., Readmond B.X., Keane V., Feigelman S., de Waard R. (2007). A double-blind, placebo-controlled, pilot study of bovine lactoferrin supplementation in bottle-fed infants. J. Pediatr. Gastroenterol. Nutr..

[48] Nguyen D.N., Li Y., Sangild P.T., Bering S.B., Chatterton D.E.W. (2014). Effects of bovine lactoferrin on the immature porcine intestine. Br. J. Nutr..

[49] Godfrey K.M., Reynolds R.M., Prescott S.L., Nyirenda M., Jaddoe W.V., Eriksson J.G., Broekman B.F.P. (2017). Influence of maternal obesity on the long-term health of offspring. Lancet Diabetes Endocrinol..

[50] Fields D.A., Schneider C.R., Pavela G. (2016). A narrative review of the associations between six bioactive components in breast milk and infant adiposity. Obesity.

